# Children’s comprehension monitoring of multiple situational dimensions of a narrative

**DOI:** 10.1007/s11145-015-9568-x

**Published:** 2015-05-22

**Authors:** Stephanie I. Wassenburg, Katinka Beker, Paul van den Broek, Menno van der Schoot

**Affiliations:** Department of Educational Neuroscience and LEARN! Research Institute for Learning and Education, Faculty of Psychology and Education, VU University Amsterdam, van der Boechorststraat 1, 1081 BT Amsterdam, The Netherlands; Department of Education and Child Studies and the Brain and Education Lab, Faculty of Social and Behavioural Sciences, Leiden University, Leiden, The Netherlands

**Keywords:** Comprehension monitoring, Situation model, Dimensions, Inconsistency detection, Validation, Availability

## Abstract

Narratives typically consist of information on multiple aspects of a situation. In order to successfully create a coherent representation of the described situation, readers are required to monitor all these situational dimensions during reading. However, little is known about whether these dimensions differ in the ease with which they can be monitored. In the present study, we examined whether children in Grades 4 and 6 monitor four different dimensions (i.e., emotion, causation, time, and space) during reading, using a self-paced reading task containing inconsistencies. Furthermore, to explore what causes failure in inconsistency detection, we differentiated between monitoring processes related to availability and validation of information by manipulating the distance between two pieces of conflicting information. The results indicated that the monitoring processes varied as a function of dimension. Children were able to validate emotional and causal information when it was still active in working memory, but this was not the case for temporal and spatial information. When context and target information were more distant from each other, only emotionally charged information remained available for further monitoring processes. These findings show that the influence of different situational dimensions should be taken into account when studying children’s reading comprehension.

## Introduction

To successfully comprehend a narrative, readers carefully monitor what is going on in a story along different situational dimensions (Zwaan & Radvansky, 1998). A multidimensional mental representation, or situation model, of what the text is about is generated by active engagement in constructive and integrative processes during reading (Graesser, Singer, & Trabasso, 1994; Kintsch, 1988; van den Broek, Young, Tzeng, & Linderholm, 1999). Comprehension skills develop at a young age, independently from and simultaneously with basic decoding skills (Bus & van IJzendoorn, 1999; Kendeou, van den Broek, White, & Lynch, 2009; Oakhill & Cain, 2012; Whitehurst & Lonigan, 1998). The present study focused on children’s ability to index events over the course of the narrative, extending previous research in two ways. First, we measured children’s monitoring skills with respect to different situational dimensions within the same paradigm. Second, within every situational dimension we differentiated between availability and validation of relevant text information, to investigate what causes comprehension difficulties. That is, we sought to uncover whether comprehension monitoring difficulties might be related to problems constructing or reactivating material (i.e., availability) and/or problems detecting inconsistencies (i.e., validation).

## Theoretical framework

During construction of a situation model, there is a continual interaction between textual information and world knowledge (Kintsch, 1986). Both are integrated into the model, which can be modified later when it is updated with new incoming information (Glenberg, Meyer, & Lindem, 1987). To maintain a coherent representation throughout the text, readers constantly have to evaluate their understanding and regulate their reading processes and strategies. This monitoring of comprehension is an important component skill of reading comprehension (Cain, Oakhill, & Bryant, 2004; Rubman & Waters, 2000). It involves detecting inconsistencies between incoming information and earlier encountered information or prior knowledge and using comprehension repair strategies if needed (van der Schoot, Reijntjes, & van Lieshout, 2012). For this reason, the *inconsistency paradigm* is often used to assess comprehension monitoring (e.g., Albrecht & O’Brien, 1993; Long & Chong, 2001).

In this paradigm, participants read passages, some of which contain a coherence break, caused by information that contradicts earlier information. For example, participants read about a characteristic of the protagonist (e.g., Mary is a strict vegetarian). A few sentences later in the story an action that is inconsistent with the information about the protagonist is described (e.g., She ordered a cheeseburger and fries; Albrecht & O’Brien, 1993). The increase in processing difficulty for inconsistent information is reflected in the inconsistency effect: information that is inconsistent with earlier context information yields longer reading times than consistent information (Albrecht & O’Brien, 1993; Hyönä, Lorch, & Rinck, 2003; Rinck, Hähnel, & Becker, 2001). Increases in reading times are hereby taken as an indication that new text information, in the perception of the reader, contradicts available previous information. According to the situation-model theory (Zwaan & Radvansky, 1998), inconsistencies specifically impede the updating process, during which a new sentence or clause is incorporated into the integrated situation model. Obviously, “new incoming information is more difficult to integrate into the evolving model if it is inconsistent, or less consistent, with the information in the current state of the model” (van der Schoot et al., 2012, p. 1667).

## Comprehension monitoring

Children often fail to successfully monitor their comprehension (e.g., Markman, 1979; Orrantia, Múñez, & Tarín, 2014; Vosniadou, Pearson, & Rogers, 1988). Studies have shown that children with reading comprehension difficulties perform worse than children without comprehension difficulties on tasks in which they have to deal with inconsistencies in a text (e.g., August, Flavell, & Clift, 1984; Ehrlich, Remond, & Tardieu, 1999; Oakhill, Hartt, & Samols, 2005; Yuill & Oakhill, 1991). Nevertheless, as pointed out by van der Schoot et al. (2012), there is no consensus about the locus of this effect. From the literature, there are two possible hypotheses that may underlie comprehension monitoring difficulties. To be able to detect an inconsistency the child must have the prior information that leads to an inconsistency available in working memory (i.e., the context information; Gerrig & O’Brien, 2005). Therefore, one hypothesis is that children fail to detect an inconsistency because the prior information is not *available*. This could be due to problems with either construction or reactivation of a rich situation model. An inconsistency will simply go unnoticed when the information that is contradicted is not available, even when children are able to engage in further updating and restoring processes (Albrecht & O’Brien, 1993; Hyönä et al., 2003; Rinck et al., 2001; Rubman & Waters, 2000). Another hypothesis is that children find difficulty especially in *validating* the incoming information against prior text or background knowledge, which entails inconsistency detection (Isberner & Richter, 2013; Singer, 2013). That is, even when context information or relevant knowledge is readily available in working memory or the situation-model representation, children do not monitor discourse consistency or engage in comparison processes (Elliott-Faust & Pressley, 1986; Long & Chong, 2001).

To determine whether the monitoring failures in fifth- and sixth-grade students are due to lack of availability of the context information or rather to difficulties with validating,^1^ van der Schoot et al. (2012) used a reading task containing inconsistencies (e.g., Albrecht & O’Brien, 1993; Long & Chong, 2001). In their version of the task, both consistency (consistent vs. inconsistent) and the location of the inconsistent target information were manipulated. Here, target information refers to the information of interest which is either consistent or inconsistent with specific prior text (i.e., context information). In the local condition context information and the inconsistent target information were in adjacent sentences, whereas in the global condition the sentences containing the context and target information were separated by a long filler passage (from now on these conditions will be called ‘near’ and ‘distant’ condition respectively). The purpose of the filler passage was to ensure that the context information was no longer active in working memory when the participant read the target information. Thus, in order to detect inconsistencies in the distant condition, readers need to activate prior information from their situation model, which is assumed to be stored in long-term working memory (Ericsson & Kintsch, 1995; Was & Woltz, 2007), before they can validate new incoming information. An inconsistency effect (i.e., the optimal response) in the distant condition reflects increased processing difficulty for inconsistent information, due to processes related to availability of context information and validation of new information against it. In the near condition, however, context information is still readily available in working memory. Therefore, the reader only has to validate new incoming information in order to detect the inconsistency. From this, we can assume that an inconsistency effect in the near condition reflects increased processing difficulty of inconsistent information only due to validation processes. It is important to note that these processing difficulties could either reflect automatic processes that are not under the control of the reader (e.g., spreading of activation to related information), or strategic inconsistency detection processes, in which the reader deliberately tries to integrate the information with the situation model (Gerrig & O’Brien, 2005). Both cases would result in an increase in reading times.

Van der Schoot et al. (2012) have investigated potential causes of children’s difficulties with inconsistency detection in more detail. Their results indicated that, whereas good comprehenders showed an inconsistency effect in both the near and distant condition, poor comprehenders only showed this effect in the near condition. Following the above line of reasoning, they interpreted this result as evidence that poor comprehenders did not have relevant information available in the distant condition, either because they did not build situation-relevant information into their situation model or because readers failed to reactivate relevant information from the model, decreasing the likelihood that they would notice the distant inconsistencies. Importantly, because poor comprehenders showed an inconsistency effect in the near condition, an explanation in terms of impaired validation abilities could be ruled out.

## Situational dimensions

Most studies of comprehension monitoring using the inconsistency paradigm have focused on one particular situational aspect and studied whether or not that specific dimension was represented in the situation model during reading. For example, some studies focused on inconsistencies in character information (Albrecht & O’Brien, 1993; de Vega, Díaz, & León, 1997; van der Schoot et al., 2012), emotional information (e.g., Gernsbacher, Goldsmith, & Robertson, 1992), temporal information (Rinck et al., 2001) or spatial information (de Vega, 1995; O’Brien & Albrecht, 1992). These studies revealed that adult readers (see van der Schoot et al., 2012, for comparable results with children) were sensitive to inconsistencies within these dimensions, which indicates that these dimensions were routinely represented during comprehension. A rich narrative text (and the situation model constructed from it), however, typically consists of information on many different aspects of a situation. In the context of reading a narrative, the situation model must be simultaneously monitored and updated along all these different dimensions (see also the event-indexing model: Zwaan, Langston, & Graesser, 1995; Zwaan, Radvansky, Hilliard, & Curiel, 1998; Zwaan & Radvansky, 1998).

In their chapter on multidimensionality, Therriault and Rinck (2007) briefly reviewed the rationale for each situational dimension of a situation model and, more interestingly, indicated the importance of exploring their relative dominance. The vast amount of literature confirms the significance of each of the different types of situational dimensions for constructing and updating situation models. It could be argued, however, that there are differences in the extent to which readers spontaneously monitor the different dimensions (Zwaan et al., 1998). Empirical support for this assumption comes from various lines of research. For example, it has been shown that, in order to establish and maintain coherence during narrative reading, it is particularly helpful to understand the causal relations (Bohn-Gettler, Rapp, Van den Broek, Kendeou, & White, 2011; Rapp, van den Broek, McMaster, Kendeou, & Espin, 2007; van den Broek, 1994). It has even been indicated that readers monitor causal relations routinely (Singer, 2006). When thinking about cause and effect of certain events (i.e., causal information), but also the emotions of the protagonist (i.e., emotional information), our thoughts seem more concrete and imageable than, for example, when thinking about the temporal order of events or the time span of a certain situation (i.e., temporal information) (Burgoon, Henderson, & Markman, 2013; Evans, 2007). Unlike causal events and emotions, time is not a concrete sensory experience. Spatial information can be seen as either concrete or abstract. The spatial dimension is often represented as a description of a specific setting or location, which is concrete and imageable. Spatial information, however, can also represent the three-dimensionality of things. In this sense, it is experienced as a more abstract dimension. Moreover, emotion and causation are described as second-order dimensions (Therriault & Rinck, 2007), in that emotion is intrinsically related to intentionality and causation, and causation is intrinsically related to time. Supposedly, this relatedness to, or even dependency on, other situation-model information also makes the dimensions of causation and emotion more comprehensive. As a consequence, a situation model constructed from more comprehensive information is presumably richer than the situation model constructed from less comprehensive information. If emotional and causal information are indeed more comprehensive than, for example, temporal and spatial information, it may be useful, particularly for readers with limited capacities like children, to prioritize monitoring these more comprehensive types of information (Bohn-Gettler et al., 2011). For the present purposes, it is important to note that there are no studies that take the multidimensional character of situation models into account when studying comprehension monitoring abilities in primary school children by means of the inconsistency paradigm.

In a comparative study of adults and middle-school children, Bohn-Gettler et al. (2011) investigated comprehension monitoring by looking at reading times for sentences containing shifts in situational dimensions. It is the first study so far to examine children’s monitoring skills on different dimensions within a single paradigm. The results showed that, whereas adults are able to spontaneously monitor all situational dimensions (i.e., characters and goals, causation, time, and space), children’s reading times only increased at causal shifts. This result may indicate that children tend to focus on the most dominant features during the processing of text information (Piaget & Inhelder, 2000). Supposedly, causality is the most dominant situational dimension [see for example Taylor and Tversky (1997) index dominance hypothesis] and children’s restricted monitoring abilities cause them to focus on this particular dimension. Whereas Bohn-Gettler’s study provides more insight into differences between children’s and adults’ monitoring skills on different situational dimensions, it does not differentiate between component processes of comprehension monitoring (e.g., availability vs. validation related processes). Furthermore, their texts contained shifts in more than one dimension. Research shows, however, that shifts in situational dimensions (and the way they are processed) are correlated with each other (Therriault & Rinck, 2007). That is, monitoring information in one dimension influences the monitoring of information in another if the text contains shifts–or, for that matter, inconsistencies–in both dimensions. Hence, when studies use texts containing shifts in more than one dimension it is impossible to draw valid conclusions about inherent differences between situational dimensions.

## The present study

In order to explore whether previous findings of processes related to information availability and validation in children could be extended to other situational dimensions (i.e., emotion, causation, time, and space), we constructed a reading task containing inconsistencies as used by van der Schoot et al. (2012). For this purpose, we adopted their line of reasoning according to the situation-model framework. This means that to detect an inconsistency, relevant information should be available to validate new incoming information against it. Detecting distant inconsistencies, therefore, requires both activation and validation processes, whereas detecting near inconsistencies (where information is still active in working memory) only reflects the validation process. For the reading task, short narrative passages were constructed in which the target information was either consistent or inconsistent with regard to the context information presented earlier in the passage. In the near condition the two sentences immediately followed each other, whereas in the distant condition the two were separated by a filler paragraph. In the present study, each passage was designed to have one specific situational dimension as most dominant. Context and target information always contained information based on this particular dimension, while other sentences in the passage were held as dimension-neutral as possible. Accordingly, each passage contained only one inconsistency in either emotional, causal, temporal or spatial information. This way, we were able to investigate whether children’s monitoring skills differ as a function of type of (contradictory) information, while ruling out the influence of dimension dominance. On the basis of previous findings (Bohn-Gettler et al., 2011; Rapp et al., 2007), we expected that children do not process all situational dimensions in the same way. We tentatively hypothesized that children find it easier to deal with inconsistencies on the dimensions of emotion and causation than on the dimensions of time and space. This hypothesis was based on the assumptions that the former dimensions are more comprehensive than the latter, that they are more concrete (Burgoon et al., 2013; Evans, 2007), and that they are more entwined with other dimensions (Therriault & Rinck, 2007).

To explore whether potential differences in monitoring skills were due to differences between the situational dimensions rather than to differences between other properties of the passages, it was necessary to make sure that the relative dominance of the dimension of interest within its specific context was equal for all texts. In line with the above, relative dominance here reflects how significant the contradictory information is within a specific passage. To do so, we presented the passages to expert adults and asked them to rate the passages by answering the question “How important do you think the contradictory information (context and target sentence) is for a global understanding of what the whole text was about?” on a five-point Likert scale. In other words, when the contradictory information is rated as important, removing it would change the meaning of the passage as a whole. Moreover, in order to acquire converging evidence for the hypothesis that temporal and spatial inconsistencies are more difficult to detect than emotional and causal inconsistencies, the experts were also asked to estimate how difficult they thought it would be for children to detect the inconsistency.

Finally, if children would show difficulties with dealing with inconsistencies along certain situational dimensions, reading time patterns for near and distant inconsistencies would provide more insight into processes related to the availability of information and validation.

## Method

### Participants

The participants were 192 children (85 boys) in Grade 4 (*n* = 87) and Grade 6 (*n* = 105) from four regular primary schools in different areas of average to high socio-economic status in the Netherlands. The children were 8–13 years of age (*M* = 10.77, *SD* = 1.08). All children came from schools with relatively high concentrations of native Dutch students and were fluent Dutch speakers. Only children who did not have (diagnosed) behavioral problems or learning disabilities as indicated by the school records participated. Decoding skills were measured by the EMT, a standardized Dutch word reading test (Brus & Voeten, 1999). Students’ raw EMT scores were recoded into their respective normally distributed norm scale (0–10). Both fourth-grade (*M* = 5.16, *SD* = 1.75) and sixth-grade (*M* = 5.36, *SD* = 1.81) students had grade-level decoding skills and there was no difference between grades with regard to their norm-related performance, *t*(189) = −.59, *p* = .555. In accordance with a procedure preferred by schools and endorsed by the ethical committee of the faculty, parents were provided a letter about the aim and procedures of the study and could respond by returning a preprinted objection note. Participation was voluntary and children received a small gift after the experiment.

### Materials and design

#### Reading task

All passages had the same structure and were designed to be adequate for students of all reading levels (i.e., Grade 4 reading level and higher). In the first sentence a situation was introduced, for example, “The children got their grades for the English test.” The second sentence was always the context sentence, for example, “Steve had studied very hard for the test and got a F/an A.” This sentence, relative to the later target sentence, provided key context information in one of the four situational dimensions (i.e., emotion, causation, time, or space). The sentence of interest (target sentence), for example, “Steve was very happy and couldn’t wait to tell his parents”, contained information that was either consistent or inconsistent with regard to the context information (respectively “Steve… got an A” vs. “Steve… got a F”). In the near condition, the target sentence immediately followed the context sentence. In the distant condition, a dimension-neutral filler paragraph of four sentences (e.g., about how the teacher had marked the tests) preceded the critical target sentence. The purpose of the filler paragraph was to make sure the context information was no longer active in working memory by the time readers encountered the target sentence (Albrecht & O’Brien, 1993; Long & Chong, 2001). Whether the target information was consistent or inconsistent depended on the context information. The context sentence in the consistent condition differed in only a few words from the context sentence in the inconsistent condition. Within each dimension, the number of syllables for the context as well as the target sentences was approximately the same for all passages. Target sentences were exactly the same in all versions (i.e., near/consistent, near/inconsistent, distant/consistent and distant/inconsistent) of a passage, to increase comparability between conditions. Passages ended with one wrap-up sentence following the target sentence, for example, “After school, Steve ran home to tell his mom”. See the “Appendix” for the complete set of experimental passages.

After each passage, a simple dimension-neutral question appeared on the screen, requiring a yes/no answer. This was to give the participants a reading goal and to ensure they would take the task seriously. To allow for generalization of results, texts contained a variety of information within each dimension. For example, in the temporal dimension inconsistencies could be based on: age differences, temporally bound events, order of events, length of time typically needed for events, etc. (Rinck et al., 2001). Passages were presented in a Courier New-type font, with a 18 point size.

In total, there were 16 within-subjects conditions formed by crossing three factors: consistency (consistent vs. inconsistent), location (near vs. distant), and situational dimension (emotion, causation, time, and space). Each participant was presented with 32 experimental passages, 2 in each condition. In other words, for every dimension, eight different passages were constructed, with four versions of every passage (i.e., near/consistent, near/inconsistent, distant/consistent and distant/inconsistent). The stimuli were arranged into four lists, each containing the 32 passages. In order to ensure full combination of conditions and materials, the four different versions of each passage within each dimension were counterbalanced across the lists by means of a Latin square design. Each list was presented to approximately the same number of children. Thus, across lists and across participants, each passage within each dimension occurred equally often in the near/consistent, near/inconsistent, distant/consistent and distant/inconsistent version. Passages within each list were presented in a pseudo randomized order.

#### Questionnaire

Thirty-five expert adults (i.e., university employees with a master’s degree in either psychology or education) were asked to read one of the four material lists. For each of the 32 passages, they indicated whether it was consistent or inconsistent (context and target sentences were highlighted). Only when they indicated the information as inconsistent, two additional questions had to be answered. First, participants were asked to indicate on a five-point Likert scale how dominant they thought the highlighted information (context and target sentence) was within that specific passage (“How important do you think the highlighted information is for a global understanding of what the whole text was about?”). Second, participants were asked to indicate on a five-point Likert scale how difficult they thought the inconsistency would be to detect by the children in our sample.

### Self-paced moving window method

Reading times were collected using the self-paced moving window method (Just, Carpenter, & Woolley, 1982). This means that passages were presented on a computer screen, but were masked by X’s. By pressing the down-arrow key on the keyboard, a window moved down the text and revealed one line at a time, whilst previous sentences were masked again. By pressing the up-arrow key, the window moved up to reveal the previous sentence again. Only test leaders were allowed to use this key if necessary (e.g., when a child needed to read the instructions again). Participants were instructed not to read back to previous sentences. Key pressing latencies (i.e., reading times) correspond to the period a sentence was first displayed until the next key press and reflect the online processing of the sentence, in line with the eye-mind assumption (Just & Carpenter, 1980).

Note that looking back or re-reading are important comprehension monitoring strategies, reflecting double-checking or repairing comprehension. In this study, however, we aimed to investigate processes related to availability and validation of information which are necessary for detecting an inconsistency, rather than more high-level strategic processes that are executed after detecting the inconsistency in order to restore comprehension. Furthermore, van der Schoot et al. (2012) conducted an additional experiment, using an eye-tracker device, after their self-paced reading experiment to investigate look-backs and found that it was highly unlikely for both good and poor comprehending primary school children to look back and reread the target information.

### Procedure

All participants were tested individually in a private room at their own school. They sat at a computer and completed the experiment at their own pace. First, children were informed that the study was designed to investigate reading comprehension of short narratives presented on the screen. They were instructed to read silently at a normal pace, like they would do when reading a book at home, and to comprehend the passages as accurately as possible because they would be asked a question about each passage afterwards to check their understanding. No information was provided with regard to inconsistencies. Children were under the assumption they would read ‘normal’ passages. Next, a short text appeared on the computer screen explaining the self-paced reading method to the participants. The experimenter checked whether they had correctly understood the instructions and procedure. All passages were presented successively. Every passage was preceded by the line ‘NEW STORY’. During presentation of this line participants were allowed to pause for a moment when needed. They resumed reading with the down-arrow key. Each sentence of a passage consisted of either one or two lines of text. After each passage was completely read, a question was presented in the moving window (i.e., these never concerned information from the context or target sentence). Participants answered by saying yes or no to the experimenter. To prevent participants from getting bored or tired, the task was paused after 16 texts. They could take a few minutes break during this pause. After participants had completed the reading task, they were thanked for their participation and returned to their classroom. In total, the experiment took approximately 25–40 min.

## Results

### Reading task

Analysis of the accuracy scores showed that responses on the passage questions were above chance (*M* = 90 % correct, range 72–100 %), indicating that the children did not guess. The high overall accuracy suggests that participants were attentive to stories which lends confidence to the further analyses.

During the experiment, children were sometimes distracted or started talking to the experimenter during presentation of the target sentence. In order to remove data reflecting non-reading delays, reading times of >3 *SD*s from the overall condition mean were excluded. Furthermore, extremely short reading times of <80 ms per syllable were taken as an indication that the reader had accidentally pressed the space bar too soon and did not read the sentence completely, and were therefore excluded from the analyses. In total, this resulted in removal of less than 5 % of the reading times.

**Fig. 1:**
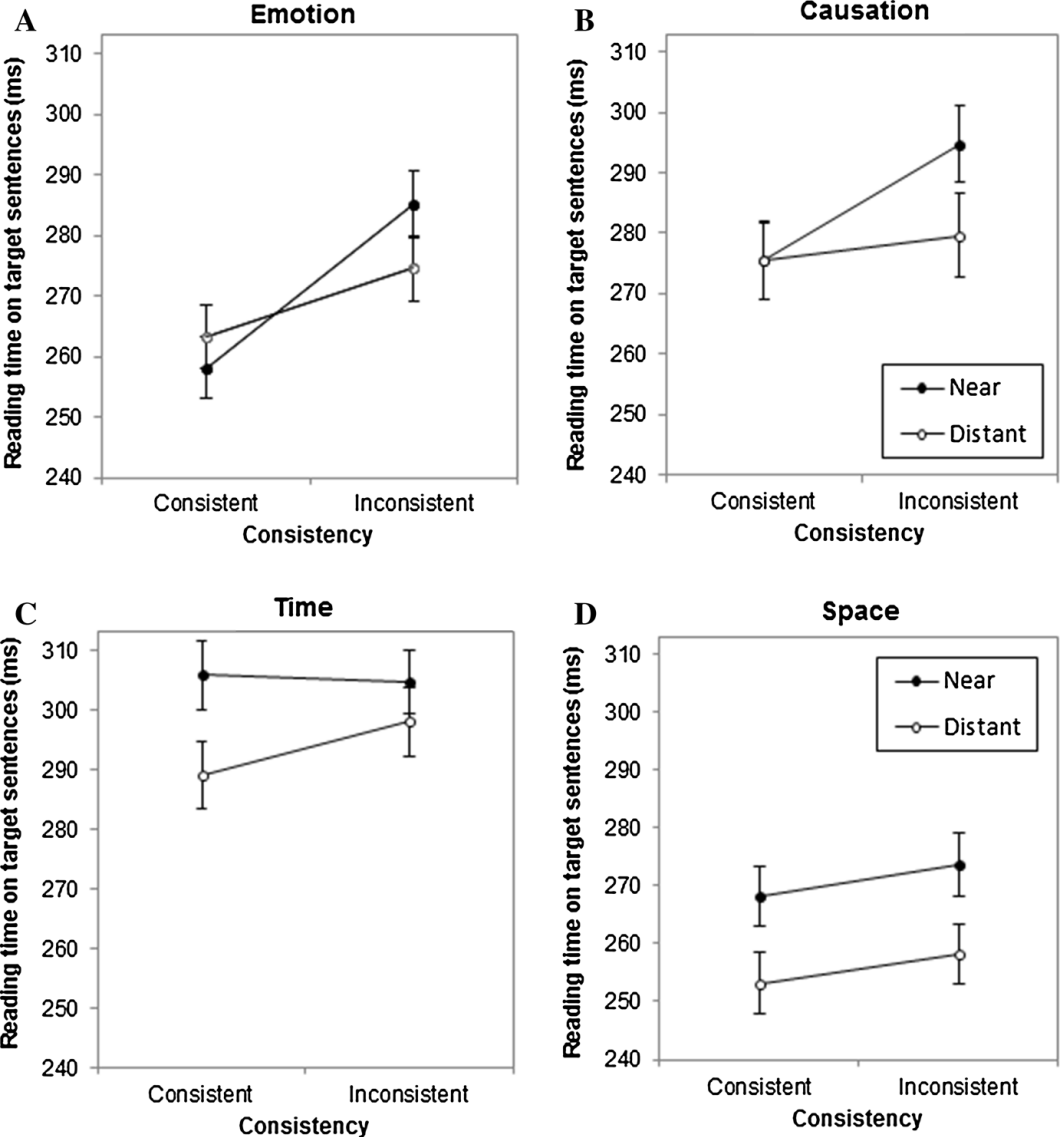
Reading times per syllable on target sentences (in ms) as a function of consistency and location for (**a**) emotional, (**b**) causal, (**c**) temporal and (**d**) spatial information. *Error bars* depict standard errors of the means

First, overall 2 × 2 × 4 mixed analyses of variance (ANOVA) were conducted on the reading times. Consistency (consistent vs. inconsistent), location (near vs. distant) and dimension (emotion, causation, time, and space) were included as within-subject factors.^2^ Due to the limited number of items per condition only analyses with variability due to subjects were performed. Second, to further explore the influence of dimension on inconsistency detection additional 2 × 2 ANOVAs were conducted for every dimension with location and consistency as within-subject factors. In Fig. 1, reading times on the target sentence (in milliseconds) are presented as a function of consistency, location and dimension. Counterbalancing list was included as a between-subject factor, because it interacted significantly with consistency and dimension (Pollatsek & Well, 1995). However, effects for the list variable are not reported given the lack of theoretical relevance. Furthermore, for all comparisons that included dimension, Mauchly’s test indicated that the assumption of sphericity had been violated, *p* < .05. Therefore degrees of freedom were corrected using Greenhouse–Geisser estimates of sphericity.

The results of the overall ANOVA showed that reading times were faster in the distant than in the near condition, *F*(1, 188) = 24.64, *MSE* = 2649, *p* < .001, η_p_^2^ = .12.^3^ Furthermore, inconsistent target sentences were read more slowly than consistent target sentences, *F*(1, 188) = 29.98, *MSE* = 2489, *p* < .001, η_p_^2^ = .14, which indicates that, generally, children detected the inconsistencies.

This effect of consistency, however, differed significantly as a function of dimension, Consistency × Dimension: *F*(2.81, 527.60) = 4.46, *MSE* = 2354, *p* < .01, η_p_^2^ = .02. Thus, the type of contradictory information influenced to what extent the reading process was slowed down upon encountering an inconsistency. More specifically, inconsistent target sentences yielded longer reading times especially when the contradicted information concerned the emotional or causal aspects of a described situation. However, as can be seen from Fig. 1, these interaction effects of consistency and dimension could be attributed to children slowing down on the emotion and causation inconsistencies in the near condition, as was evident in the three-way interaction Consistency × Dimension × Location: *F*(2.88,540.58) = 4.08, *MSE* = 1965, *p* < .01; η_p_^2^ = .02.

Follow-up ANOVAs were performed to further explore the reading times patterns within each dimension separately. As indicated above, the results showed that the effect of consistency was only present for the dimensions of emotion, *F*(1, 188) = 35.36, *MSE* = 2043, *p* < .001, η_p_^2^ = .16, and causation, *F*(1, 188) = 7.50, *MSE* = 3238, *p* < .01, η_p_^2^ = .04. For the emotion dimension (see Fig. 1a), the effect of consistency was present in both the near condition, when target information immediately follows context information; *t*(191) = −6.00, *p* < .001, and the distant condition, where context and target information were separated by a filler paragraph; *t*(191) = −2.65, *p* < .01. The effect, however, was clearly larger in the former than in the latter, Consistency × Location: *F*(1, 188) = 8.44, *MSE* = 1518, *p* < .01; η_p_^2^ = .04. With regard to passages containing causation related information (Fig. 1b), inconsistent target sentences were also read slower than consistent target sentences. This effect, however, was only significantly present in the near condition; *t*(191) = −3.46, *p* < .01 and *t*(191) = −.655, *p* = .51 for the near and distant condition respectively, Consistency × Location: *F*(1, 188) = 4.46, *MSE* = 2580, *p* < .05, η_p_^2^ = .02.

For the temporal and spatial dimensions (see Fig. 1c, d respectively), no effects of consistency were evident. Inconsistent sentences did not yield longer reading times in either the near or distant condition. Surprisingly, however, in both dimensions participants speeded up their reading when a text was longer; target sentences after a filler passage (in the distant condition) were read significantly faster than target sentences which immediately followed the context sentences (in the near condition), which resulted in a main effect of location, Time: *F*(1, 188) = 11.91, *MSE* = 2150, *p* < .01, η_p_^2^ = .06; Space: *F*(1, 188) = 27.54, *MSE* = 1543, *p* < .001, η_p_^2^ = .13.

### Questionnaire

A repeated-measures ANOVA with location (near vs. distant) and dimension (emotion, causation, time, and space) as within-subject factors was conducted on accuracy scores, dominance ratings and difficulty ratings. Again, we controlled for the counterbalancing list by including this as a between-subject factor. Results indicated that location did not influence the accuracy scores, *F*(1, 31) = .44, *ns*, but dimension did, *F*(3, 93) = 23.17, *MSE* = .65, *p* < .001, η_p_^2^ = .43. Post-hoc contrast analysis revealed that accuracy was significantly lower for the temporal dimensions than for the other dimensions, *F*(1, 31) = 47.66, *MSE* = 2.47, *p* < .001, η_p_^2^ = .61.

The following analyses include only items that were correctly identified as inconsistent. As expected, results on the mean dominance ratings showed that the information in the context and target sentences was rated as more dominant in the near condition (i.e., short passages that contain nearly no other information) than in the distant condition, where passages are longer due to filler information, *F*(1, 29) = 23.84, *MSE* = 19.72, *p* < .001, η_p_^2^ = .45. More importantly, there were no differences in dominance between the different dimensions, *F*(3, 87) = .96, *ns*.

Finally, results on the mean difficulty ratings showed that expert readers expected distant inconsistencies to be harder to detect by primary school children than near inconsistencies, *F*(3, 87) = 5.43, *MSE* = 4.55, *p* < .05, η_p_^2^ = .14. Furthermore, rated difficulty was not the same for all dimensions, *F*(3, 87) = 15.81, *MSE* = 11.68, *p* < .001, η_p_^2^ = .35. Planned contrasts confirmed that spatial and temporal inconsistencies were thought to be significantly harder to detect than emotional, *F*(1, 29) = 23.65, *MSE* = 37.50, *p* < .001, η_p_^2^ = .45, and causal inconsistencies, *F*(1, 29) = 36.66, *MSE* = 31.92, *p* < .001, η_p_^2^ = .56.

## Discussion

The present study focused on the multidimensional character of a situation model. The results provide insight into children’s comprehension monitoring skills along various situational dimensions by constructing, reactivating, and updating a mental representation of a text. The obtained reading time patterns indicate that type of information is an important factor in children’s comprehension monitoring. Moreover, the pattern of results within each dimension provide insight into the underlying processes causing the failure of detecting inconsistencies in text (van der Schoot et al., 2012). In short, comprehension monitoring involves validating information against prior text and background knowledge in order to maintain a rich and coherent mental representation of the situation described in a text. Therefore, the relevant information has to be available and active. In the near condition, information is still active in working memory. In the distant condition, availability of information means the reader has constructed all relevant context information into a situation model and reactivated this if necessary. The question is which of these processes (i.e., processes related to ensuring availability or to validation) could explain children’s difficulties when dealing with texts containing inconsistencies and whether this differs with regard to type of information (i.e., emotion, causation, time, space). In the present study, we indirectly examined availability and validation of information during reading by measuring the participants’ self-paced reading times. Specifically, detecting inconsistencies in the distant condition, when context information is no longer active in working memory, would reveal availability of relevant information from a situation model and subsequently, validation of the new incoming information against it. In the near condition, however, detecting an inconsistency would only represent the validation process, because availability of the information is ensured by the short distance between the context information and the target information.

### Situational dimensions

The results suggest that children perform best on monitoring narratives containing emotional information. The reading times pattern on this particular dimension replicated the findings of van der Schoot et al. (2012) with respect to their group of good comprehenders. Children’s reading slowed down when they encountered an inconsistency based on emotional information in both the near and distant condition. This inconsistency effect was much stronger though in the near than in the distant condition. With regard to texts containing causal information, the pattern of results replicated van der Schoot’s previous findings with respect to their poor comprehenders. Children’s reading slowed down on causally inconsistent sentences in the near condition but not the distant condition. The inconsistency effect in the near condition indicated that children validated incoming information against available prior text and related background knowledge. The lack of an inconsistency effect in the distant condition, however, was interpreted as an indication that causal inconsistencies went unnoticed, because the information that was contradicted was not available (i.e., it was not constructed into the situation model or retrieved from memory). Together, the results indicate that children were able to not only validate information along the situational dimension of the protagonist (as was previously demonstrated by van der Schoot et al., 2012) but also along the dimensions of emotion and causation. In contrast to emotional and causal information, children did not seem to monitor temporal and spatial information. The results showed that there was no inconsistency effect present for the temporal and spatial passages. The reading times for these two types of passages did not differ for inconsistent and consistent target sentences in both the near and distant conditions.

### Differences between dimensions

The present results are in line with the hypothesis that emotional and causal inconsistencies are easier to detect for primary school children than temporal and spatial inconsistencies. This could be due to their dependency on or relatedness to other dimensions (Therriault & Rinck, 2007). Emotion is related to intentionality and causation, whereas causation is related to time (Stein & Levine, 1989; Therriault & Rinck, 2007). Time and space, however, are dimensions that do not necessarily depend on other types of information (Therriault & Rinck, 2007). Presumably, this makes emotional and causal information more comprehensive than temporal and spatial information and thus more helpful to establish and maintain coherence in a narrative (Rinck et al., 2001).

An alternative, but related, explanation for the differences in monitoring between the situational dimensions involves their relative processing difficulty due to differences in concreteness of situational aspects (Burgoon et al., 2013). Temporal and spatial information may be more difficult to monitor than emotional and causal information because, for example, thinking in terms of time may be less concrete or sensory-perceptual than thinking about emotions or cause-effect-relations (Evans, 2007). Developmental investigations have shown that time-related processing abilities continue to develop throughout the elementary school years (Mackinlay, Kliegel, & Mäntylä, 2009; Stein & Levine, 1989). This can be understood considering that children may be more likely to be involved in emotional experiences (e.g., compliments make you happy) or cause-effect-experiences (e.g., water extinguishes fire) than experiences associated with time perception (e.g., experiences associated with time discrimination or time assessment, such as the ability to estimate the duration of daily-life events) (Taylor & Tversky, 1997). A similar line of reasoning may be applied to the spatial dimension. Spatial information processing requires more skilled processing capabilities and, although children may be able to simply represent location, they have difficulties updating this information (Plumert, 1996). Moreover, in the present study, a spatial inconsistency was rather implicit (as were inconsistencies in other dimensions) in comparison to an inconsistency in specific location or setting. For example, an inconsistency could be caused by the direction of motion by the protagonist or by the place of objects or people relative to the protagonist (e.g., “Wilma sat at in the last row staring at her test. (…) She turned around to see the answers of her friend behind her”). In other words, detecting these inconsistencies required three-dimensional thinking, instead of remembering a specific location or setting. Research shows that adults and older children are more likely to monitor spatial information than young children (Bohn-Gettler et al., 2011; Gauvain & Rogoff, 1989). Consistent with this ‘processing difficulty’ explanation, our results are in line with previous neuroimaging studies showing that validating information and subsequently updating a situation model is a content-specific process, in that updating different types of information elicits activation in different brain areas (Ferstl, Rinck, & von Cramon, 2005). This supports the conclusion that differences in monitoring skills between dimensions are due to situational content, rather than to textual characteristics.

### Questionnaire

To obtain more insight into the features of our experimental texts and their potential influence on the obtained results, expert adult readers were asked to rate the texts on the relative dominance of the dimension of interest within a specific passage and on difficulty to detect the inconsistency for children. They indicated that the contradictory information was rated as equally dominant in all dimensions. This result is important as it suggests that our dimension effects reflect actual, inherent differences between the situational dimensions rather than differences at the level of the passages (i.e., differences between dimensions in their relative dominance to the texts in which they appear). The experts, however, indicated that they would expect differences in the ease with which children would be able to detect the inconsistencies in the different dimensions. As expected, spatial and temporal inconsistencies were thought to be harder to detect than emotional and causal inconsistencies. Interestingly, this was also reflected in the experts’ own accuracy scores which were lower for temporal inconsistencies, a finding which replicates earlier findings of Ferstl and von Cramon (2007).

The results of the questionnaire support the interpretation of the experimental results of our study. It seems that emotional and causal information are as dominant as temporal and spatial information in the present passages, but they are somehow easier to monitor. Supposedly, the former are more comprehensive than the latter because of their relatedness to other situational dimensions. Information on the dimensions of time and space does not depend on other dimension and can only contradict itself on a single aspect (i.e., a temporal inconsistency only violates coherence with regard to time, while an emotional inconsistency can violate coherence with regard to cause-effect relations or the intentions of the protagonist), which makes such a contradiction harder to detect. This explanation is supported by research showing higher error rates for temporal passages (Ferstl & von Cramon, 2007) and may account for the higher error rates for temporal inconsistency detection in our questionnaire. Also, it is consistent with findings that causal relations are crucial in establishing coherence and that they are more likely to be monitored by children than other types of information (Bohn-Gettler et al., 2011; Rapp et al., 2007). To draw firm conclusions about differences in comprehension monitoring between dimensions, it would be useful to repeat this study with more items.

### Availability and validation

Inspection of children’s reading time patterns across situational dimensions also gives insight into dimension-related differences with regard to availability-related processes and validation processes. Children were able to *validate* information against prior text and background knowledge along the dimensions of both emotion and causation. This was reflected by the inconsistency effects they displayed in the near condition, in which the context information was still active and thus readily available in working memory. The differential influence of these situational dimensions could be observed, however, with regard to *availability* of information in the distant condition. Although children were able to detect distant emotional inconsistencies, they did not seem to detect the distant causal inconsistencies.

The latter effect is consistent with previous studies showing that children have difficulties with detecting inconsistencies, especially when relevant information is no longer readily available (Long & Chong, 2001; Oakhill et al., 2005). That is, children tend to fail to reactivate this information from their situation model in long-term working memory or they leave out critical situation-relevant information from the model that could be used as a basis to interpret later text information. This leaves us with the question of how to explain the finding that children did notice the distant inconsistencies for the emotion passages. It might be possible that children became particularly engaged in comprehension processes when reading about emotional events and, therefore, raised their standards of coherence (Komeda & Kusumi, 2006; van den Broek, Risden, & Husebye-Hartmann, 1995).

### Future directions

Overall, the results clearly indicate the importance of the type of information for different components of comprehension monitoring skill. The different types of contradictory information within a situational dimension, however, should be explored in more detail (Rinck et al., 2001). It could be, for example, that not all types of temporal aspects (such as duration, order of events, etc.) are equally important to form an integrated and coherent mental representation of a text. Next, we will point to some other issues that should be taken into consideration in future research. First, in narrative texts, causal relations and information with regard to the protagonist are typically dominant. Therefore, future research should include other types of texts in order to explore their possible influence on children’s comprehension monitoring of the different situational dimensions. For example, science texts are more abstract and contain more crucial temporal and spatial information (Best, Floyd, & Mcnamara, 2008). This makes strategic monitoring and background knowledge more important, which could potentially influence children’s monitoring behavior. Second, in the present study, we did not examine potential interactive relations between situational dimensions. As mentioned before, monitoring of information in one situational dimension may affect monitoring of information in another dimension when a text contains shifts or inconsistencies along both dimensions (e.g., Therriault & Rinck, 2007). Further research should take this more into account, especially in relation to availability of information and validation. Third, we were not able to investigate why relevant information was no longer available when children encountered distant inconsistencies in the causal, temporal and spatial passages. The current results cannot differentiate between whether this was due to a failure to reactivate information from their situation model or a failure to incorporate the situation-relevant context information in their model in the first place. Further studies on children’s monitoring of different situational dimensions should try to differentiate between these possibilities (see, for example, Long & Chong, 2001). Finally, a limitation of the present study is the lack of a comprehension outcome variable for the experimental passages. The passages were too short to enable construction of meaningful comprehension questions (i.e., the ‘near’ version of a passage only contained four sentences and questions were not supposed to direct children to detecting the inconsistency), which rendered us without information on potential differences in comprehension for the different passages. In a similar vein, no control measure of general reading comprehension was included in the analyses. We recommend including measures of reading comprehension in future research. This may provide converging evidence for differences in processing difficulty between situational dimensions.

## Conclusion

In summary, the present findings extend previous research in several ways. It shows that children do not seem to monitor all situational dimensions spontaneously as adults do: emotional and causal information are supposedly easier to monitor during reading than temporal and spatial information. This may reflect the fact that emotion and causation are related to other dimensions, which makes them more comprehensive and thus more helpful in establishing and maintaining coherence and enabling the reader to understand a text at a global level. Furthermore, although children seem to be able to engage in construction, reactivation, and validation processes, they do not do this for all types of situational information to the same degree. Together, these findings suggest that including explicit focus on the different situational dimensions of a narrative in reading comprehension education may be conducive to teaching children strategic monitoring on the basis of a rich and coherent situation model.

## Appendix

Reading materials used in the present study (translated from Dutch)

**EMOTIONAL PASSAGE #1**

The children got their grades for the English test.

**Consistent context:** Steve had studied very hard for the test and got an A.

**Inconsistent context:** Steve had studied very hard for the test and got a F.

*They had done the English test yesterday.*

*Their teacher had marked them as fast as she could.*

*She had even taken the tests home.*

*At home she was better able to concentrate on marking them.*

**Target sentence:** Steve was very happy and couldn’t wait to tell his parents.

After school, he ran home to tell his mom immediately.

Q—Did the children get a grade for the English test? A—Yes

**EMOTIONAL PASSAGE #2**

Emma plays volleyball.

**Consistent context:** Her team is ranked last, because they’re the worst team in the league.

**Inconsistent context:** Her team is ranked first, because they’re the best team in the league.

*Her family often watches when she plays.*

*They cheer and encourage her from the sidelines.*

*Sometimes, someone scores in the last few seconds of the game.*

*That makes it very exciting to watch.*

**Target sentence:** Emma is disappointed in herself and also in her team.

Next week is the last game of the season.

Q—Does Emma play baseball matches? A—No

**EMOTIONAL PASSAGE #3**

Rick just got a new bike and cycled to the city center.

**Consistent context:** He forgot to lock it but luckily a few minutes later, it was still there.

**Inconsistent context:** He forgot to lock it and a few minutes later, it was already stolen.

*It was very busy in the city.*

*During the weekend there were always a lot of tourists.*

*Rick was not used to going to the city center.*

*He preferred to go on less busy days.*

**Target sentence:** Rick felt really relieved and promised to pay more attention.

He wanted to go home as fast as possible.

Q—Did Rick forget to lock his bike? A—Yes

**EMOTIONAL PASSAGE #4**

Eric walked across the school yard by himself and passed a group of girls.

**Consistent context:** He waved at the girls to say hi, but they laughed at him loudly.

**Inconsistent context:** He waved at the girls to say hi and they smiled back at him.

*Eric just came back from a visit to the dentist.*

*That was why he arrived later at school than his friends.*

*He had already missed the first two classes.*

*The next class started after recess.*

**Target sentence:** The girls made him happy and he stopped to talk to them for a while.

Eric knew a few of these girls from his music class.

Q—Did Eric walk across the school yard with his best friend? A—No

**EMOTIONAL PASSAGE #5**

Vera had a job at an office to earn some extra money.

**Consistent context:** She had to get up early in the morning and did not have much to do at work.

**Inconsistent context:** She had to get up early in the morning and had lots of things to do at work.

*Vera went to school on the other days of the week.*

*She was nearly finished with her studies.*

*At work, she shared an office with two other girls.*

*They had decorated their room to make it a bit more cozy.*

**Target sentence:** She was often bored the whole day and just talked to her colleagues.

Usually she worked 2 days a week.

Q—Did Vera work at an office? A—Yes

**EMOTIONAL PASSAGE #6**

Will got lost in the city last week.

**Consistent context:** It was already quite late in the evening and strangers helped him.

**Inconsistent context:** It was already quite late in the evening and strangers robbed him.

*Will had never been in that part of the city.*

*He was looking for his uncle’s place.*

*At home he had memorized a map.*

*He got lost somewhere anyway.*

**Target sentence:** He felt so thankful that he had run into these strangers.

It was totally dark when he finally arrived home.

Q—Did Will get lost in the city early in the morning? A—No

**EMOTIONAL PASSAGE #7**

A small country in Europe had had the same president for a long time.

**Consistent context:** The president was an old man and he was not very popular with the people.

**Inconsistent context:** The president was an old man and he was very popular with the people.

*He often had to give speeches.*

*During those speeches, people could watch him on TV and listen on the radio.*

*Usually he wore a nice suit with golden buttons.*

*His hair was grey and he was going a little bit bald.*

**Target sentence:** The people of the country disliked their president.

After 12 years, they finally chose another president.

Q—Did they choose another president after 12 years? A—Yes

**EMOTIONAL PASSAGE #8**

Marie loved to travel and her boyfriend always stayed home.

**Consistent context:** She would be leaving soon to go travelling through the US for a year.

**Inconsistent context:** She would be coming back soon from travelling through the US for a year.

*Marie had always wanted to go to the States.*

*She had saved up a lot of money for this trip.*

*She always preferred to go traveling on her own.*

*That way she could do whatever she wanted.*

**Target sentence:** Marie’s boyfriend was sad, because in a week, the time would finally arrive.

The whole family would come along to the airport.

Q—Was Marie travelling through Asia? A—No

**CAUSAL PASSAGE #1**

Sophie had moved house not long ago.

**Consistent context:** She had painted the walls white and also got a new white carpet.

**Inconsistent context:** She had painted the walls black and also got a new black carpet.

*Her parents had helped her carry a lot of heavy boxes.*

*One big moving box fit a lot of stuff.*

*It had been hard work, but it was worth it.*

*Sophie was very pleased with the result.*

**Target sentence:** Her new room appeared very light and bright.

She felt immediately at home.

Q—Did Sophie get a new carpet? A—Yes

**CAUSAL PASSAGE #2**

The boy scouts went camping in the forest and made a big campfire.

**Consistent context:** When it started to get dark, the scouts threw big logs of wood on the fire.

**Inconsistent context:** When it started to get dark, the scouts threw big buckets of water on the fire.

*The scouts had organized a party.*

*There were loads of different kinds of food and drinks.*

*They all camped in the forest and slept in tents.*

*Everyone loved to be surrounded by nature.*

**Target sentence:** The campfire grew bigger and kept everyone warm.

It turned out to be a lovely event.

Q—Was the campfire on the beach? A—No

**CAUSAL PASSAGE #3**

Maya felt like taking a long walk.

**Consistent context:** It was very cold outside, but she didn’t mind at all.

**Inconsistent context:** It was very hot outside, but she didn’t mind at all.

*Maya just liked to be out of the house.*

*Sometimes she went for a run in the park.*

*It helped her think about certain things.*

*She loved nature and everything in it.*

**Target sentence:** Maya put on her jacket and her woolen scarf.

She looked in the mirror and walked out the door.

Q—Did Maya feel like taking a walk outside? A—Yes

**CAUSAL PASSAGE #4**

Marc walked around the toyshop.

**Consistent context:** He bought balloons with his pocket money and filled the biggest one with air.

**Inconsistent context:** He bought balloons with his pocket money and filled the biggest one with water.

*He often played with balloons.*

*He bought them, especially when he was bored.*

*He particularly liked the sound they made when they popped.*

*Sometimes he could entertain himself like this for hours.*

**Target sentence:** When he let go, the large balloon slowly took off in the wind.

Marc hurried to get another balloon.

Q—Did Marc buy the balloons at the supermarket? A—No

**CAUSAL PASSAGE #5**

Joe was the first that woke up this morning.

**Consistent context:** He made himself some coffee and drank all of it.

**Inconsistent context:** He made himself some coffee and poured it into a cup.

*Joe was a bit addicted to coffee.*

*He drank it every morning before he left the house.*

*At work, he drank even more coffee.*

*The first cup at breakfast, however, was his favorite.*

**Target sentence:** The empty cup fell, when he accidentally knocked it over.

Joe had always been a bit clumsy.

Q—Did Joe make coffee for other people in the morning? A—No

**CAUSAL PASSAGE #6**

Frank had PE class twice a week.

**Consistent context:** The last time, Frank did participate well during the PE class.

**Inconsistent context:** The last time, Frank did not participate during the PE class.

*They went outside because the weather was so nice.*

*In the summer, PE classes were outside regularly.*

*Behind the school building, there was a large field.*

*They could do every sport they wanted there.*

**Target sentence:** The next day, Frank’s muscles felt sore.

His dad even had to drive him to school.

Q—Did Frank have PE classes twice a week? A—Yes

**CAUSAL PASSAGE #7**

It was always very busy at the crossing down the road.

**Consistent context:** When Kevin approached the crossing, all of the traffic lights turned red.

**Inconsistent context:** When Kevin approached the crossing, all of the traffic lights turned green.

*Sometimes Kevin would just drive around for fun.*

*He had just gotten his driver’s license.*

*He wanted to get some experience by driving around and practicing.*

*Busy crossings still made him a bit nervous.*

**Target sentence:** All of the cars in front of him stopped in a perfect line.

Today, Kevin planned on visiting his grandma.

Q—Was it busy at the crossing? A—Yes

**CAUSAL PASSAGE #8**

The student lived in a small room in the city.

**Consistent context:** The knob to turn on the heater was broken, which left it always off.

**Inconsistent context:** The knob to turn off the heater was broken, which left it always on.

*The student had been living away from his parents for 2* *years now.*

*He liked to live in the center of the city.*

*He shared his bathroom and kitchen with another student*

*His own room was small and cozy.*

**Target sentence:** The heat was almost unbearable sometimes.

There was no money to pay a mechanic in order to fix the heater.

Q—Did the student have a lot of money? A—No

**TEMPORAL PASSAGE #1**

John was meeting up with his girlfriend Sarah.

**Consistent context:** John’s train arrived 20 min later than Sarah’s train.

I**nconsistent context** John’s train arrived 20 min earlier than Sarah’s train.

*They hadn’t seen each other in a while.*

*They had lots to talk about.*

*John brought Sarah flowers and chocolate.*

*He was planning on giving the flowers to her as soon as he saw her.*

**Target sentence:** When he got off the train, Sarah was already waiting for him.

They walked downtown together.

Q—Did Sarah and John both take the train? A—Yes

**TEMPORAL PASSAGE #2**

Josh and Sam knew each other from school.

**Consistent context:** After class, Sam would first go to soccer practice before meeting Josh.

**Inconsistent context:** After class, Sam would first meet Josh before going to soccer practice.

*They often met up in the afternoon.*

*Usually they played computer games.*

*Sometimes they looked up interesting pictures for school assignments.*

*They were very skilled at finding these pictures.*

**Target sentence:** Soccer practice lasted longer than expected, so Sam was late for meeting Josh.

Josh was playing a new computer game.

Q—Did Josh and Sam know each other from soccer practice? A—No

**TEMPORAL PASSAGE #3**

Lucy and Nils were in the same class.

**Consistent context:** They usually leave for school together and prefer to walk.

**Inconsistent context:** They usually leave for school together and prefer to take the train.

*Lucy and Nils always had loads to talk about.*

*They had so much fun together.*

*They had been in the same class for years now.*

*They knew each other very well.*

**Target sentence:** This morning, Lucy decided to cycle, so she was a lot faster.

Their first course this morning was history.

Q—Did Lucy cycle to school this morning? A—Yes

**TEMPORAL PASSAGE #4**

Gus was on his way to the movies.

**Consistent context:** They were showing a long animated movie he hadn’t seen before

**Inconsistent context:** They were showing a short animated movie he hadn’t seen before.

*Gus loved movies about art and culture.*

*But he also liked action movies.*

*He had already seen most of them.*

*He went to the movies quite often.*

**Target sentence:** The movie was beautiful and 3 h later Gus walked out of the movie theatre.

He couldn’t stop thinking about it for a while.

Q—Did Gus leave the cinema after 1 h? A—No

**TEMPORAL PASSAGE #5**

Everyone in class was on time for a change.

**Consistent context:** A nice history teacher was going to teach the class until 10 o’clock.

**Inconsistent context:** A nice history teacher was going to teach the class starting at 10 o’clock.

*The children also had a math class today.*

*Math was pretty difficult.*

*They needed a calculator and they were going to use formulas.*

*History was easy, you only had to listen.*

**Target sentence:** When their teacher was still talking at 10.30, the class got agitated.

There was a short break after class.

Q—Was everyone in class on time? A—Yes

**TEMPORAL PASSAGE #6**

Luke went to work by car every morning.

**Consistent context:** This morning he decided to take the highway and drove very fast.

**Inconsistent context:** This morning he decided to take the highway and got stuck in a traffic jam.

*Luke had to get up early each day.*

*He didn’t really mind though.*

*Luke was a morning person and liked going to bed early.*

*He enjoyed the fresh morning air.*

**Target sentence:** Today Luke arrived at work even earlier than usual.

He had an important meeting that day.

Q—Did Luke take the highway each morning? A—No

**TEMPORAL PASSAGE #7**

Vincent and Chris are brothers.

**Consistent context:** Chris is 4 years younger than Vincent and they also have a little sister.

**Inconsistent context:** Vincent is 4 years younger than Chris and they also have a little sister.

*Their little sister is a newborn baby.*

*The neighbors visit sometimes to take a look at her.*

*She’s not able to talk yet.*

*Everybody likes to cuddle her.*

**Target sentence:** Lately, Vincent feels that he is too mature to play with Chris.

That’s why he prefers to hang out with the neighbors’ kid.

Q—Does Vincent like to hang out with neighbors’ kid? A—Yes

**TEMPORAL PASSAGE #8**

Eve and Anton were having breakfast.

**Consistent context:** Anton ate yoghurt and prepared a sandwich for school, Eve only drank milk.

**Inconsistent context:** Eva ate yoghurt and prepared a sandwich for school, Anton only drank milk.

*The breakfast table was located in a large kitchen.*

*The kitchen was brand new.*

*Everything was well maintained and clean.*

*Through the kitchen window, they could see the sun shining.*

**Target sentence:** Eve finished breakfast first and had to wait for Anton.

A moment later, they left for school together.

Q—Did Anton and Eve eat toast for breakfast? A—No

**SPATIAL PASSAGE #1**

Two blocks away, an old man was living in a big house.

**Consistent context:** Construction workers were renovating the attic of the house.

**Inconsistent context:** Construction workers were renovating the basement of the house.

*The old man wanted to turn it into a game room.*

*He had already bought a pool table.*

*His grandchildren loved playing pool.*

*They were very excited to play it.*

**Target sentence:** The old man went upstairs to bring the construction workers some drinks.

He took the empty glasses back to the kitchen.

Q—Did the old man live in a big house? A—Yes

**SPATIAL PASSAGE #2**

Jim had a cat named Remy.

**Consistent context:** One day the cat was locked outside the house and was looking for a way in.

**Inconsistent context:** One day the cat was locked inside the house and was looking for a way out.

*The cat was pacing back and forth.*

*He had tried everything.*

*He got tired of trying to come up with a solution.*

*He couldn’t think of what to try next.*

**Target sentence:** The cat couldn’t go inside, no matter how hard he scratched the door.

Angrily, he walked away.

Q—Was the cat’s name Jim? A—No

**SPATIAL PASSAGE #3**

Dan had trouble deciding what sport he wanted to do.

**Consistent context:** Recently he had started something new: trampoline lessons.

**Inconsistent context:** Recently he had started something new: scuba diving.

*His friends were all very curious.*

*They wanted to go and watch him do that.*

*Dan became very good at it in a short period of time.*

*He tried his best and practiced a lot.*

**Target sentence:** When he first started, he wasn’t even able to go higher than 3 m.

According to the instructor, Dan was a natural.

Q—Did Dan find it hard to decide on a new sport? A—Yes

**SPATIAL PASSAGE #4**

When Marly came home after school, her mother was still at work.

**Consistent context:** She waited for her mom with a cup of tea in the living room.

**Inconsistent context:** She waited for her mom with a cup of tea at the kitchen table.

*Her mom worked very often and was very busy.*

*Marly often sang some happy songs while she was waiting.*

*Sometimes she just stared out the window.*

*Through the window she could see her mom arriving.*

**Target sentence:** Marly got hungry and walked to the kitchen to make a sandwich.

Her mom came home after Marly had finished her sandwich.

Q—Did Marly come back from work? A—No

**SPATIAL PASSAGE #5**

Ali and Ron were playing catch together.

**Consistent context:** Ron tried to catch the ball, but he missed and it landed just behind him.

**Inconsistent context:** Ron tried to catch the ball, but he missed and it landed in front of him.

*The boys played on the sidewalk.*

*They were right beside a busy road.*

*They had to make sure that the ball would not roll into the road.*

*Ron wasn’t very good at catching the ball.*

**Target sentence:** He turned around quickly to get the ball before it would roll away.

As hard as he could, he threw the ball back to Ali.

Q—Were Ali and Ron playing soccer? A—No

**SPATIAL PASSAGE #6**

Kyle was going to meet his friends at a bar.

**Consistent context:** He looked through the window to see if they had saved a seat for him.

**Inconsistent context:** He looked through the window to see if they were arriving yet.

*They usually met at the same time.*

*The bar was not far from Kyle’s house.*

*That’s why he was often there first.*

*Usually he ordered a beer before the others came.*

**Target sentence:** Kyle went inside and heard a nice song on the radio.

He started dancing to the music.

Q—Was Kyle going to meet his friends in a bar? A—Yes

**SPATIAL PASSAGE #7**

Kelly went to see her favorite band perform.

**Consistent context:** She stood all the way in the front, in front of 5000 people watching the stage.

**Inconsistent context:** She stood all the way in the back, behind 5000 people watching the stage.

*Kelly had been a fan for years.*

*Maybe even as long as she could remember.*

*She knew all the lyrics by heart.*

*The lead singer was her favorite because he was handsome.*

**Target sentence:** She threw a teddy bear, which landed near the lead singer’s feet.

She danced and sang all night long.

Q—Did the teddy bear land on stage? A—Yes

**SPATIAL PASSAGE #8**

Wilma was not well prepared for the exam.

**Consistent context:** She was sitting at a table in the first row and was staring at her test.

**Inconsistent context:** She was sitting at a table in the last row and was staring at her test.

*She was thinking about the next day.*

*It was her birthday tomorrow.*

*She was going to shop for a gift with her mother this afternoon.*

*She already knew what she wanted.*

**Target sentence:** When the teacher looked away, Wilma turned to see her friend’s answers behind her.

Quickly, she wrote down the right answer.

Q—Did Wilma prepare well for the exam? A—No

## Footnotes

1 Labelled ‘construction’ and ‘updating’, respectively, in van der Schoot et al. (2012).

2 To explore developmental trends, the analyses were originally conducted with Grade included as between-subject factor. Although sixth graders generally read faster than fourth graders, *F*(1, 184) = 35.79, *MSE* = 1907,834, *p* < .001, η_p_^2^ = .16, Grade did not seem to confound any of the other factors, which was evidenced by the non-significant interactions, *F*s(1, 184) <2.52, *ns*. Therefore, the ANOVA was recalculated excluding the between-subjects factor of Grade.

3 0.01 is considered a small partial eta-squared effect size, 0.06 is considered a medium effect, and 0.14 is considered a large effect (Stevens, 2009).
